# 580. Pharmacokinetics (PK) and Safety of Nirmatrelvir/Ritonavir (NMV/r) in Non-Hospitalized Symptomatic Pediatric Patients Ages 6 Years and Older with COVID-19 who are at Increased Risk of Progression to Severe Disease (EPIC-Peds)

**DOI:** 10.1093/ofid/ofae631.018

**Published:** 2025-01-29

**Authors:** Jacqueline Gerhart, Heidi Leister-Tebbe, Namita Singh, Phylinda L S Chan, Haihong Shi, William D H Carey, Jean Yan, Mary Lynn Baniecki, Victoria Hendrick, Wayne Wisemandle, Ravi Shankar P Singh, Jennifer Hammond

**Affiliations:** Pfizer, Collegeville, PA; Pfizer Inc, Collegeville, Pennsylvania; Pfizer, Collegeville, PA; Pfizer, Collegeville, PA; Pfizer, Collegeville, PA; Pfizer, Collegeville, PA; Pfizer Inc., Sanford, Florida, USA, Sanford, Florida; Pfizer Inc, Collegeville, Pennsylvania; Pfizer, Collegeville, PA; Pfizer Inc, Collegeville, Pennsylvania; Pfizer, Collegeville, PA; Pfizer Inc, Collegeville, Pennsylvania

## Abstract

**Background:**

NMV/r is an antiviral treatment for mild-to-moderate COVID-19 approved for high-risk adults and authorized for high-risk adolescents ≥ 12 years weighing ≥ 40 kg under Emergency Use Authorization. EPIC-Peds evaluates PK, safety, and efficacy in pediatrics. Here we report data in patients ≥ 6 years.

**Figure:**
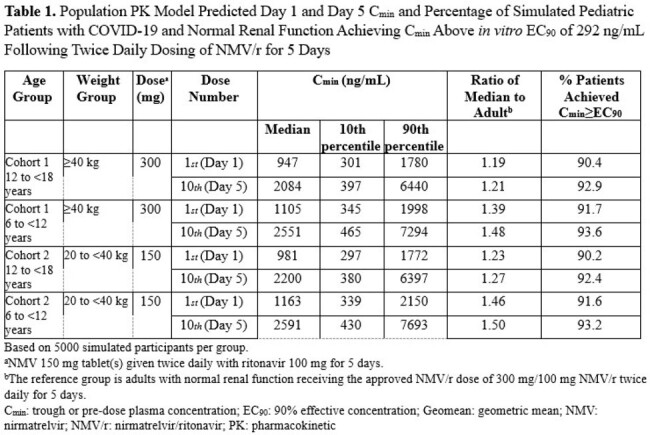

**Methods:**

EPIC-Peds is an interventional Phase 2/3 open-label study. NMV/r was administered orally twice daily (BID) for 5 days to 75 participants ≥ 6 years weighing ≥ 40 kg (Cohort 1) and weighing ≥ 20 kg to < 40 kg (Cohort 2) with symptomatic COVID-19 who were at risk of progression to severe disease. Efficacy was extrapolated from adults by matching pediatric exposure to adult COVID-19 patients receiving the indicated NMV/r dose. Sparse PK samples were collected on treatment Day 1 and 5 and incorporated into a previously developed Population PK model, which was used to simulate pediatric exposure. Safety was evaluated through Day 34. Supportive efficacy endpoints included COVID-19 related hospitalization, all-cause death, and change in SARS-CoV-2 nasopharyngeal (NP) RNA concentration from baseline to end of treatment.

**Results:**

NMV/r was safe and well-tolerated in pediatric participants. Overall, 26.7% of participants reported an all-causality adverse event (AE), with diarrhea and headache the most frequently reported in 3 (4%) participants each. AEs were mostly mild or moderate in severity, nonserious, and unrelated to treatment. Seven treatment-related AEs were reported, and none led to treatment discontinuation. No treatment-related serious AEs were reported. Simulated pediatric exposure of NMV/r of 300 mg/100 mg BID for Cohort 1 and 150 mg/100 mg BID for Cohort 2 showed > 90% of subjects achieving trough concentration (C_min_) above the 90% effective concentration (EC_90_) after the first dose and at steady state (Table 1). There were no COVID-19 related hospitalizations or all-cause deaths. The adjusted mean decrease in SARS-CoV-2 NP RNA concentration from baseline to end of treatment was ≥ -3.4 log_10_ copies/mL, numerically greater than in adults.

**Conclusion:**

In pediatric patients ages 6 years and older, 300 mg/100 mg BID and 150 mg/100 mg BID NMV/r dosing for patients weighing ≥ 40 kg and ≥ 20 to < 40 kg, respectively, was safe and effective, with NMV C_min_ > EC_90_ in > 90% of the population for both groups.

**Disclosures:**

**Jacqueline Gerhart, PhD, MBA, MS**, Pfizer: Employee|Pfizer: Stocks/Bonds (Public Company) **Heidi Leister-Tebbe, BSN**, Pfizer Inc: Employee|Pfizer Inc: Stocks/Bonds (Public Company) **Namita Singh, MD**, Pfizer: Employee|Pfizer: Stocks/Bonds (Public Company) **Phylinda L. S. Chan, PhD**, Pfizer: Stocks/Bonds (Public Company) **Haihong Shi, MS, MBA**, Pfizer: Employee|Pfizer: Stocks/Bonds (Public Company) **William D H Carey, N/A, MBBS, PhD, MRCPI**, Pfizer: Employee with stock options|Pfizer: Stocks/Bonds (Public Company) **Jean Yan, M.S.**, Pfizer: Employee|Pfizer: Stocks/Bonds (Public Company) **Mary Lynn Baniecki, PhD**, Pfizer: salaried employee|Pfizer: Stocks/Bonds (Public Company) **Victoria Hendrick, BSc**, Pfizer: Stocks/Bonds (Private Company)|Pfizer: Stocks/Bonds (Public Company) **Wayne Wisemandle, MA**, Pfizer: Employee of Pfizer|Pfizer: Stocks/Bonds (Public Company) **Ravi Shankar P. Singh, PhD, FCP**, EMD Serono: Employer of Spouse|Pfizer Inc.: Employee|Pfizer Inc.: Stocks/Bonds (Public Company) **Jennifer Hammond, PhD**, Pfizer, Inc: Employee|Pfizer, Inc: Stocks/Bonds (Public Company)

